# Development of an IoT Architecture Based on a Deep Neural Network against Cyber Attacks for Automated Guided Vehicles

**DOI:** 10.3390/s21248467

**Published:** 2021-12-18

**Authors:** Mahmoud Elsisi, Minh-Quang Tran

**Affiliations:** 1 Industry 4.0 Implementation Center, Center for Cyber-Physical System Innovation, National Taiwan University of Science and Technology, Taipei 10607, Taiwan; 2 Department of Electrical Engineering, Faculty of Engineering at Shoubra, Benha University, Cairo 11629, Egypt; 3 Department of Mechanical Engineering, Thai Nguyen University of Technology, 3/2 Street, Tich Luong Ward, Thai Nguyen 24000, Vietnam

**Keywords:** automated guided vehicle, deep learning, Industry 4.0, IoT, online monitoring, cybersecurity

## Abstract

This paper introduces an integrated IoT architecture to handle the problem of cyber attacks based on a developed deep neural network (DNN) with a rectified linear unit in order to provide reliable and secure online monitoring for automated guided vehicles (AGVs). The developed IoT architecture based on a DNN introduces a new approach for the online monitoring of AGVs against cyber attacks with a cheap and easy implementation instead of the traditional cyber attack detection schemes in the literature. The proposed DNN is trained based on experimental AGV data that represent the real state of the AGV and different types of cyber attacks including a random attack, ramp attack, pulse attack, and sinusoidal attack that is injected by the attacker into the internet network. The proposed DNN is compared with different deep learning and machine learning algorithms such as a one dimension convolutional neural network (1D-CNN), a supported vector machine model (SVM), random forest, extreme gradient boosting (XGBoost), and a decision tree for greater validation. Furthermore, the proposed IoT architecture based on a DNN can provide an effective detection for the AGV status with an excellent accuracy of 96.77% that is significantly greater than the accuracy based on the traditional schemes. The AGV status based on the proposed IoT architecture with a DNN is visualized by an advanced IoT platform named CONTACT Elements for IoT. Different test scenarios with a practical setup of an AGV with IoT are carried out to emphasize the performance of the suggested IoT architecture based on a DNN. The results approve the usefulness of the proposed IoT to provide effective cybersecurity for data visualization and tracking of the AGV status that enhances decision-making and improves industrial productivity.

## 1. Introduction

In recent industry applications following the Industry 4.0 revolution, the control of automated guided vehicles (AGVs) has triggered the utilization of remote online monitoring platforms, which are important for improving the industrial environment and increasing the speed of production processing. Typically, AGVs are employed to transport specified materials or products in several places (e.g., port terminals, automated plants, and airports) [1,2,3,4,5]. The system security of such AGVs represents the biggest challenge against online monitoring [6,7]. The main target of online monitoring is to respond rapidly to urgent problems of the operating states of the physical assets and manufacturing processes. Normally, when a propensity toward a machinery fault or failure is detected, highly experienced machine operators are capable of performing appropriate actions to prevent the outage situation of the production system.

Recently, many research works have been devoted to introducing reliable Internet of Things (IoT) infrastructures to monitor and track AGVs [8,9]. In [10], intelligent manufacturing and an IoT cloud platform were conducted by Wan et al. for advanced material handling to improve the automation capabilities with a low cost. Context-aware cloud robotics (CACRs) were used to enhance the decision-making mechanisms for the material handling processes regarding energy efficiency and cost saving. In this architecture, all robots could share the data through the cloud directly or indirectly. The cloud scheduler enabled an effective and easy strategy for the manipulation platforms and robotics to share and interact with the information. This scheduler was devoted to analyzing the location of all the robots according to the minimum element of stack vectors to perform the material handling requirements. The applied CACRs performed an analysis of the collected data from all devices in detail according to the industrial environmental conditions. The big data-based analytics and the collected information demonstrated a knowledge library between the cloud robots for learning purposes and a failure diagnosis. However, this IoT architecture did not take into account the issue of cyber attacks. In [11], a developed smart system was introduced for optimizing the scheduling of AGVs and monitoring the conditions by Yao et al. In this system, a smart combination between the data analysis and digital twin models was performed to provide smart optimized scheduling for real-time AGV management operations in complex manufacturing environments. The measured signal was conducted with an appropriate action based on a smart algorithm. However, the developed system did not take into account the issues of a failure diagnosis, time delays, data loss, and cyber attacks. The cyber attacks that occurred during the remote monitoring of the smart system increased the unmatched disturbances for the physical controlled system [12,13]. Anomalies and intrusion detection in industrial control systems (ICS) were studied based on different strategies. A variety of comprehensive surveys are devoted to the classification of strategies and methodologies on this issue [14]. The popular approach to intrusion detection in ICS is primarily based on modeling and a simulation of the machine [15,16]. Realistic problems with this method are the requirement of a specific know-how of the designs and configurations of the device as well as the complicated physical behavior of the systems. In keeping with Mitchell et al. [17], ICS anomaly detection methods consist of information- and behavior-based methods.

Information-based detection techniques search for recognized attack behaviors similar to malware signature techniques in information technology (IT) intrusion detection. Even with low false rates, these processes require the retention of an up-to-date dictionary of attack signatures and are ineffective against zero-day attacks. In assessments, behavior-based strategies search for anomalies in the runtime conduct. These strategies are more commonplace in ICS intrusion detection because ICS systems are automated and provide greater regularity and predictability than standard IT structures. Therefore, the determination of cyber attack signals based on machine learning or deep learning and the diminishing the effect of these signals by a robust model predictive control would be a key contributor to a smart manufacturing system. In [18], a new infrastructure was introduced based on artificial intelligence to validate and check the reading of smart meters to inspect if the reading was a real reading or fake. The fake reading was due to cyber attacks and ineffectual meters. Environmental factors such as temperature, humidity, and noise signals affected the performance of the meters. Moreover, the introduced infrastructure investigated the data loss that occurred through the communication channels and an unstable internet network. A classical machine learning algorithm named decision tree was devoted to handling the regression and classification of the reading of the meters. The reading visualization was performed by a developed industrial platform named CONTACT Elements for IoT. From an Industry 4.0 perspective, the security of online systems is considered to be a challenging task in the context of condition monitoring [19,20]. Appropriate action on the operating states of physical assets and manufacturing processes is needed for the suppression of cyber attacks.

This paper proposes an IoT architecture for the reliable and secure online visualization and tracking of an AGV status based on a deep neural network (DNN). The automatic identification of cyber attacks is the first step in designing a smart system for fake data suppression. The further development of real-time cyber attack monitoring needs to include a fake data suppression strategy to be a smart system in which the monitoring system could read the AGV microcontroller information, collect and analyze the sensor data, and send a control command to the automatic control interface. The superior accuracy rate of the proposed DNN is illustrated in comparison with other deep learning and machine learning algorithms in the literature. Using an innovative IoT platform called CONTACT Elements for IoT, the AGV state is visualized depending on the suggested IoT architecture. The proposed IoT architecture based on the DNN effectively identifies and simulates the normal state of the AGV. In exchange, the planned IoT architecture detects the out of service condition and essentially tracks and displays it on the dashboard of the IoT platform and creates an alert to notify the customer of the failure of the AGV. Most interestingly, the proposed IoT architecture built on the DNN easily detects network instability due to cyber attacks. To validate the feasibility of the suggested IoT platform based on the DNN, various test scenarios with a realistic configuration of AGVs and the IoT are conducted. The proposed IoT imagines and monitors the status of the AGVs, allowing for better decision-making and increased efficiency in the workplace.

## 2. Proposed IoT Architecture

The new features of internet networks and cloud computing introduce a fast and dynamic infrastructure to improve the performance of the system especially in critical and large-scale systems that utilize feedback control and the IoT [21]. The fast-acting nature of cloud computing due to its high speed and salient features leads to its use in industrial control applications. Cloud computing can overcome many problems related to physical systems [22]. Recently, IoT services including hardware and software have been made available by many information technology and industrial companies. Furthermore, the provided IoT platforms include data analytics solutions for various types of industrial systems [23]. The IoT platforms can collect and receive local sensor measurements in order to provide different processes such as storage purposes, visualization, and data analysis. The signals are transmitted between the AGV sensors and the controller by using serial communication protocols such as Modbus; the collected data are then transferred to the IoT platform via various network protocols such as HTTP and MQTT. Today, various IoT platforms are utilized for data analysis and visualization processes within distributed computing systems and cloud services. Real-time monitoring is influenced by the data collection method. Therefore, an offline data analysis is preferred for a high latency manner.

The data pre-processing includes different procedures such as data cleaning, data integration, data reduction, and data transformation. Time delay assessments, data loss estimations, and labeling procedures can also be involved in the data pre-processing stage. Following data pre-processing, the preparation and design of the data analysis model represent the main challenges to provide accurate results due to the different environmental characteristics for every factory. In this paper, the suggested IoT infrastructure is implemented based on distributed devices to provide a parallel computing process to decrease Wi-Fi and time delay issues. A database server is utilized to receive the collected data from all machines because the sensors have different communication protocols. The deep learning algorithm is then utilized through the gateway to classify the transferred data between the machines and the IoT platform in order to provide a reliable system.

The integration of the IoT into Industry 4.0 can increase the data transfer between wide and different smart devices and controllers for various applications such as domestic and industrial usage [24,25]. Indeed, the improvement of the IoT via smart microcontrollers has been implemented via prominent network telecommunications, e.g., Bluetooth and GSM, for remotely inspecting and monitoring microcontrollers. With the progress in Industry 4.0 and the IoT as well as 4G and 5G networks, AGV systems have utilized these technologies to provide a good analysis and monitoring of different datasets between users and smart systems. Thus, different architectures and platforms are utilized for specific applications based on various technologies within the Industry 4.0 trend. The diversity of IoT technologies can increase the data security risks in communication networks between connected devices. This is a hot issue in the application of IoT technology in the global industrial sector.

**Figure 1:**
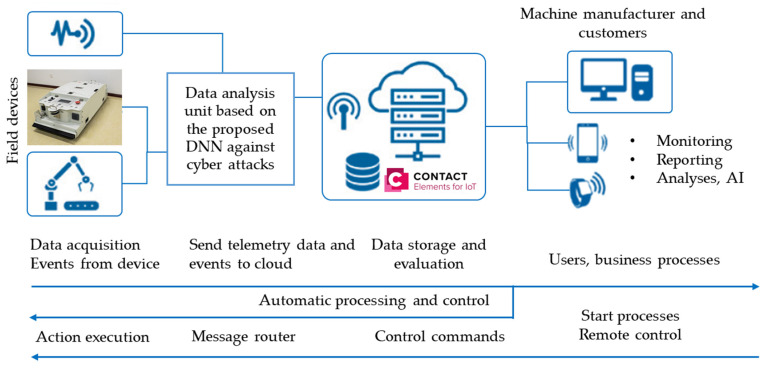
Schematic diagram of the infrastructure of IoT processing for the AGV.

In this work, the benefit of using IoT-based deep learning for the AGV was to provide reliable online monitoring, which can support decision-making in various features such as prediction, real-time visualization, remote controlling, and cyber-physical security. Figure 1 shows the CONTACT Elements for the proposed IoT that were utilized for users to interact with the IoT platform. Thus, the implementation of IoT technology was expected to provide an ease of operation, simplify supervision, enable rapid problem-solving, and increase work efficiency and effectiveness in large-scale manufacturing. In this paper, an additional unit was coupled between the sensors and the IoT platform to analyze the transferred data based on the proposed DNN against cyber attacks, as shown in Figure 1.

## 3. Deep Learning Overview

Deep learning is the one type of machine learning that tries to mimic the functionality of the neurons in the human brain. Deep learning utilizes multi-layered artificial neural networks and uses a large amount of data to automatically extract the relevant features and learn the pattern within the dataset [26]. It has shown a good performance with extremely high accuracy in many applications including speech recognition, disease detection, speed translation, and object detection. As deep learning models normally use multiple hidden layers and a large amount of data, they require high end machines that have powerful computing capabilities.

**Figure 2:**
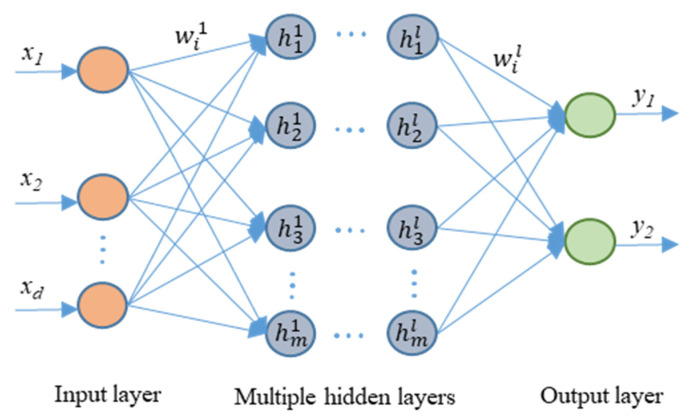
Deep neural network architecture.

Thanks to the development of technology, many cloud computing services as well as developed high performance CPUs and GPUs can perform such a large amount of multiplication operations. Recently, deep neural networks (DNNs), an artificial neural network, have become a popular network architecture. They are widely utilized to deal with classification and recognition problems. Figure 2 illustrates a structure of a DNN. Generally, the DNN comprises an input layer, an output layer, and multiple hidden layers. The input layer is defined by $X=\left[x,\;x_{1},\;\ldots x_{d}\right]$ and the output layer is represented by $Y=\left[y_{1},\;y_{1},\;\ldots y_{n}\right]$. The number of neurons of the *l*th hidden layer is *m* and $h^{l}=\left[h_{1}^{l},\;h_{2}^{l},\;\ldots h_{m}^{l}\right]$, in which each artificial neuron connects to the other that is associated with a weight and a threshold. The mapping connection of neuron *i* in the *l*th layer is described in Equation (1):

(1) $$h_{i}^{l}=f_{i}^{l}\left(w_{i}^{l}\cdot h_{i}^{l-1}+b_{i}^{l}\right)$$

where $f_{i}^{l}$ is the activation function of the neuron $h_{i}^{l-1}$, $w_{i}^{l}$ represents the vector of the weights for the connection between the neurons of layer (*l* − 1) and the *l*th layer, and $b_{i}^{l}$ describes the bias parameter of neuron *i* in the *l*th layer. Typical activation functions are the sigmoid function, the hyperbolic tangent function, or the rectified linear unit. In this architecture design, the rectified linear unit (ReLU) was used as an activation function to represent a smooth approximation, as shown in Figure 2 [27].

(2) f(x)=ln[1+exp(x)].

**Figure 3:**
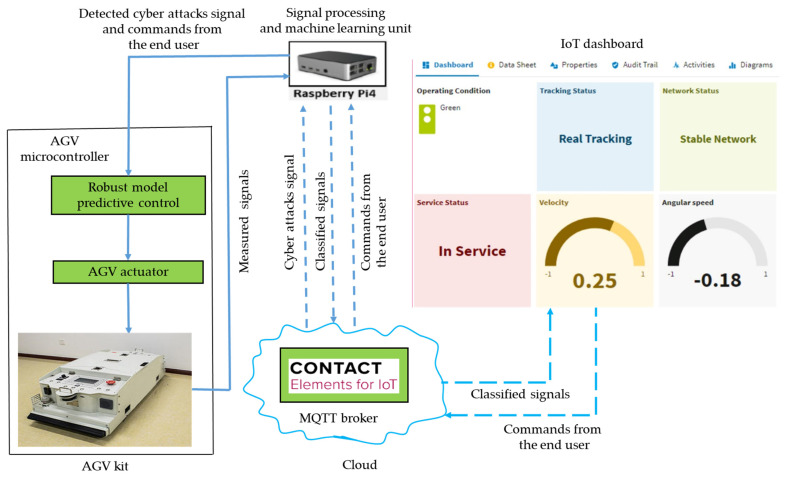
The full strategy for AGV visualization through the IoT dashboard.

The input data goes through the network and it is assigned to an estimated label at the output neuron using the softmax function, which is formulated as follows:

(3) $$y_{j}=\frac{\exp\left(h_{s,j}\right)}{\sum_{j=1}^{n_{hs}}\exp\left(h_{s,j}\right)}$$

where *h_s_* is the output of the last hidden layer and *n* represents the number of neurons at the output layer. In the training process, each iteration goes through the dataset and compares the output with the cost function. The cost function can then be reduced by adjusting the weights between the neurons using a gradient descent [28]. Figure 3 shows the full strategy of the AGV visualization through the IoT dashboard. Firstly, the ROS processed the data from LiDAR to allow the robot to automatically track a scheduled trajectory. The Raspberry Pi published the feedback signals such as the location, heading direction, and motor speed signals. The single-board computer Raspberry Pi ran on a free operating system (OS) known as the Linux-kernel operating system. Python, which has advanced as an open-source programming language, was used as the coding language to send instructions to the Raspberry Pi. A Python program was developed that allowed the Raspberry Pi to collect the data in real-time.

## 4. Results and Discussion

**Figure 4:**
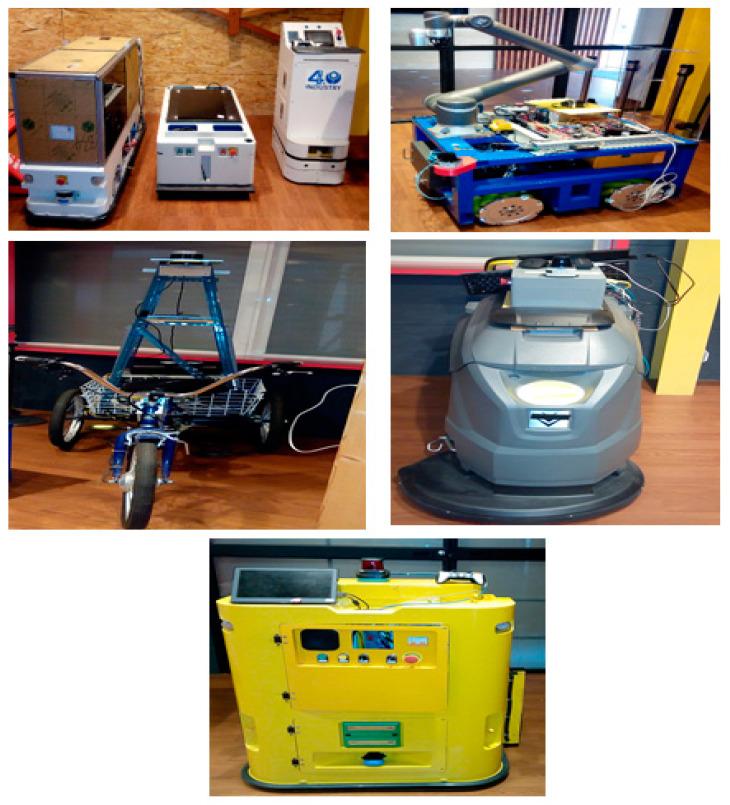
Real AGVs in the Industry 4.0 Implementation Center.

**Figure 5:**
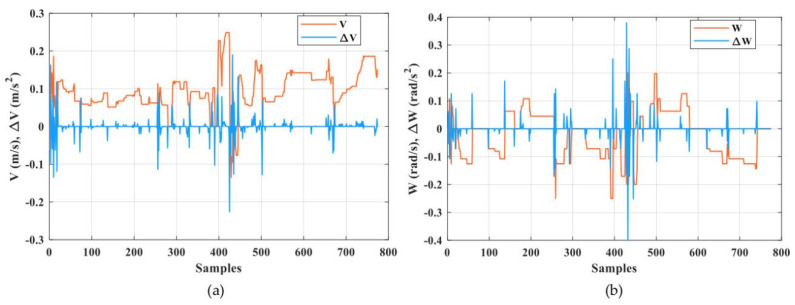
Speed input datasets of the AGV and the corresponding real value classifications. (**a**) Real data of longitudinal speed and (**b**) real data of rotational speed.

**Figure 6:**
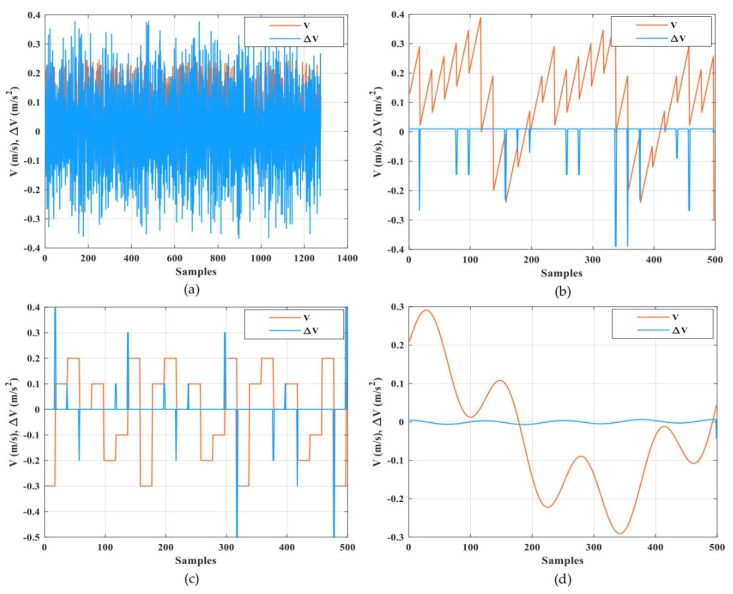
Longitudinal speed input datasets of the AGV and the corresponding fake value classifications. (**a**) Random fake data; (**b**) ramp fake data; (**c**) pulse fake data; and (**d**) sinusoidal fake data.

**Figure 7:**
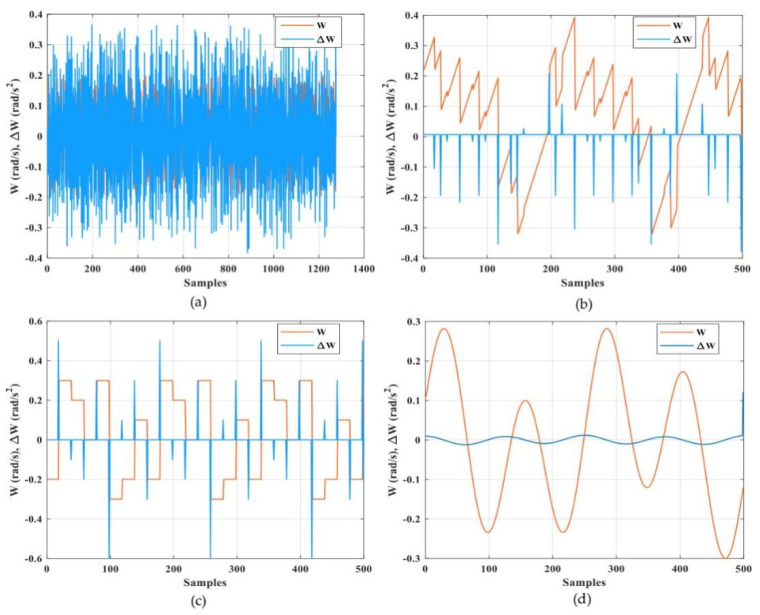
Rotational speed input datasets of the AGV and the corresponding fake value classifications. (**a**) Random fake data; (**b**) ramp fake data; (**c**) pulse fake data; and (**d**) sinusoidal fake data.

Automated guided vehicles predicate on one or various computer-controlled wheels that work without the necessity for an onboard user or driver. AGVs have predefined tracks or regions within the plant that they can navigate. Navigation is performed in different ways; for example, laser guidance, optical strips, or surface-mounted magnets. Figure 4 shows a few examples of AGVs that are utilized for experiments in the Industry 4.0 Implementation Center, Center for Cyber-Physical System Innovation, National Taiwan University of Science and Technology. Above AGVs or any other devices in the Industry 4.0 Implementation Center are smart devices and they are connected with IoT platforms. Any device of this cyber-physical system can be exposed to cyber attacks. Cyber attackers can harness this susceptibility and occupy dominance of a single device, a section of a system, or the complete system and inspire substantial harm; for example, service perturbation, data loss, and device damage. A smart attacker can access the transmitted signals between the AGV and the IoT platform easily by manipulating these measured or received signals via internet networks. As a result, the system performance may degrade and force the system to operate at non-economical operating conditions due to non-optimal control signals or even lead to instability. There are different types of cyber attacks against numerical signals such as scaling attacks, ramp attacks, pulse attacks, and random attacks [29,30]. A scaling attack changes the true measurement signals to higher or lower values based on a scaling factor and a ramp attack changes the true measurement signals by the addition of a ramp factor. A pulse attack modifies the true measurement signals by adding spaced short pulses and a random attack increases or decreases the true measurement signals in a random manner. It is necessary to apply a suitable technique to recognize these types of attacks to overcome the problems due to them [31]. In this paper, the proposed DNN was trained and tested with experimental data from the AGV that represented the real status, as shown in Figure 5. The proposed DNN was also trained and tested with other types of attack data such as a random attack, ramp attack, pulse attack, and sinusoidal attack to account for the worst cases of attacks that represent the fake status, as shown in Figure 6 and Figure 7. The proposed DNN was devoted to recognizing the transferred data of the AGV. The data of the AGV consisted of two classes named “Real Tracking” and “Fake Tracking”. Real tracking appeared when the transferred data was real and the internet network was stable. Fake tracking appeared when there was a cyber attack on the internet network that sent fake data. Further test scenarios were performed to confirm the effectiveness of the proposed IoT architecture based on the DNN. The steps of the proposed IoT architecture based on the DNN were concluded in Algorithm 1, which had the pseudo-code of the suggested strategy.

### 4.1. Dataset

In order to recognize cyber attacks to enhance the reliability and security of the online visualization and tracking of the AGV status, experiments were conducted to collect the AGV information. An autonomous cleaning robot received the spiral path navigation and it began to track the trajectory until it reached the final destination. At the same time, the Raspberry Pi published the robot positions and robot conditions. The collected data included the velocity and the rotation speed, which were collected from a real AGV and labeled as 0. The fake data that equated to the cyber attack was created by randomly distributed functions within the range of the AGV signals and labeled as 1. Both the real data and fake data were combined to form one dataset. The collected dataset had 3563 samples, which were split into 2850 samples for the training process and 713 samples for testing. All datasets were normalized before feeding into the defined network. In this case, the min–max approach was applied for the normalization, as shown in Equation (4):

(4) $$x^{\prime }=\frac{x-\min\left(x\right)}{\max\left(x\right)-\min\left(x\right)}.$$

The DNN model was designed to consist of four hidden layers. The details of the model parameters in each layer are shown in Table 1. Figure 4, Figure 5 and Figure 6 show the speed input datasets of the AGV and the corresponding real or fake value classifications as well as the longitudinal and rotational speed (*V*, *W*) and the change of longitudinal and rotational speed (Δ*V*, Δ*W*).
**Algorithm 1. The steps of the proposed IoT architecture based on the DNN****1: Read** the speed signals from AGV microcontroller.**2: Connect** to the MQTT broker.**3: Input** data to the DNN model.**4: Recognize** the AGV status by the DNN.**5: If** the result of DNN = = 0.**6: Publish** that the AGV status is ‘Real Tracking’ and the network status is ‘Stable Network’.**7: Else if** the result of the DNN = = 1.**8: Publish** that the AGV status is ‘Fake Tracking’ and the network status is ‘Unstable Network’.**9: If** the velocity > 0.**10: Publish** that the service status of the AGV is ‘In Service’.**11: Else**.**12: Publish** that the service status of the AGV is ‘Out of Service’.**13: End**.

### 4.2. Performance Assessment

**Figure 8:**
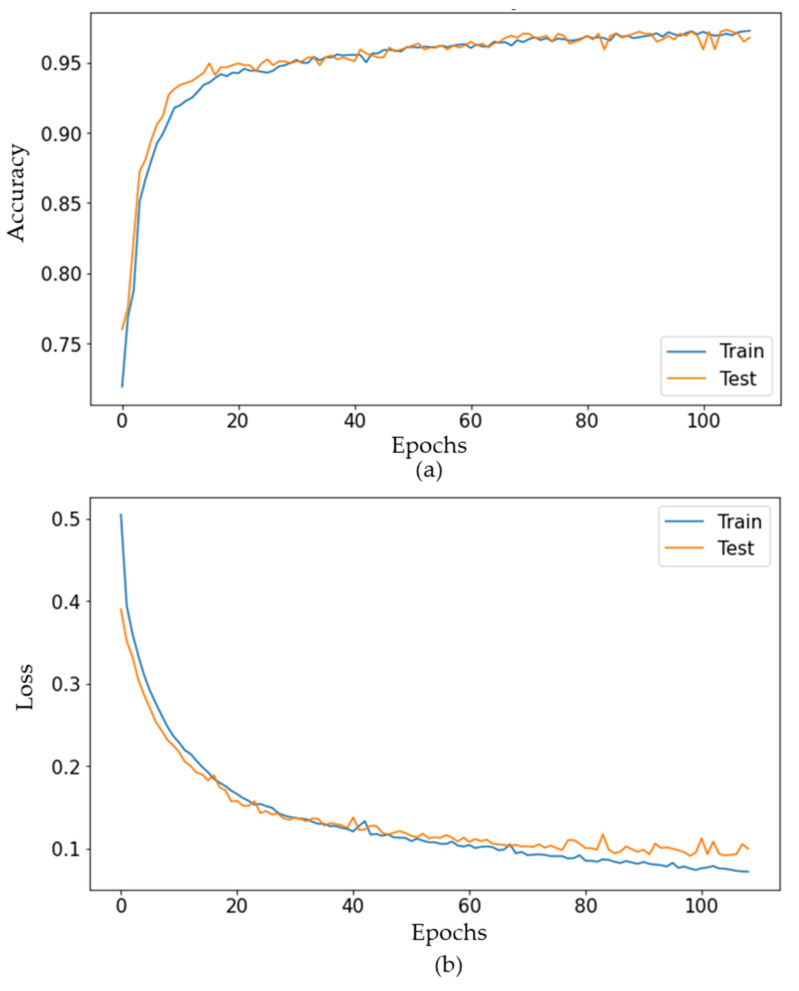
The performance of the proposed deep neural network. (**a**) Model accuracy and (**b**) model loss.

The DNN model was built based on the prepared training dataset and testing dataset to predict the status of the AGV in which the number of epochs was set to 150. A batch size of 32 was used as the default setting and 4 hidden layers were implemented with the defined parameters described in Table 1. Moreover, early stopping criteria and dropout techniques were included in the training model to suppress the overfitting problem and to improve the performance of the proposed DNN model. The other hyperparameters were set to default values for a fair comparison with other machine learning models. Figure 8 illustrates the performance of the proposed deep neural network for the cyber attack detection of the AGV. It shows that both the training and testing processes could quickly reach higher than 95% accuracies after 20 epochs. Finally, the DNN model resulted in excellent accuracy with approximately 97.25% for the training and 96.77% for the testing after 110 epochs. Figure 8b illustrates the training loss and testing loss, in which both values continuously decreased. The testing loss value was marginally higher than the training loss value at the stable point. It indicated a good fit of the model without overfitting.

**Figure 9:**
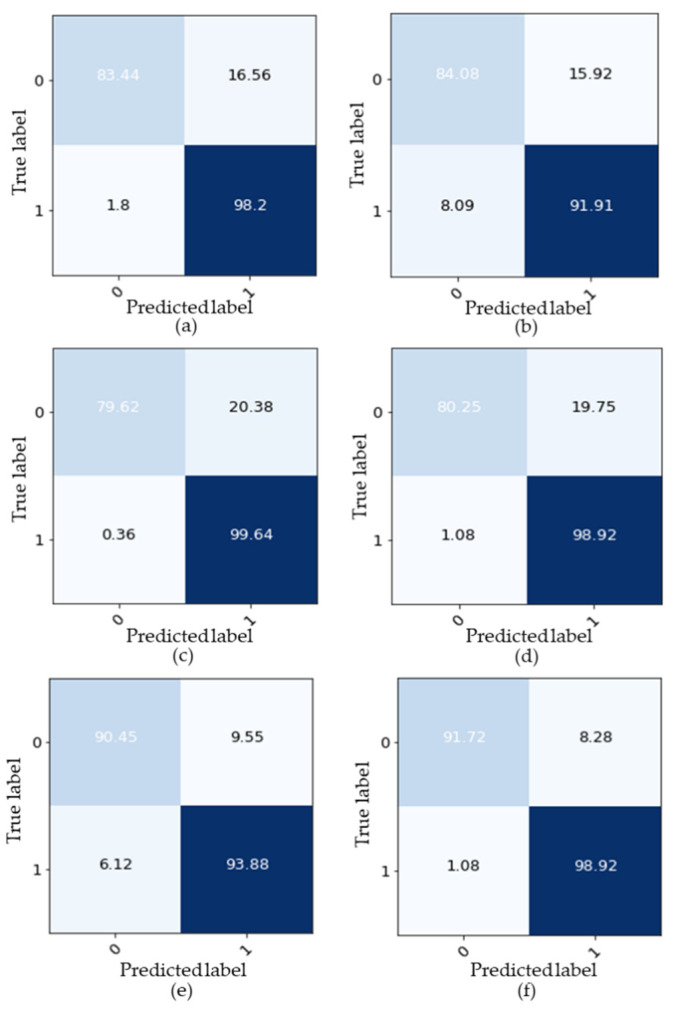
Diagnosis confusion matrix “Real data label = 0, Fake data label = 1”. (**a**) Decision tree model; (**b**) SVM model; (**c**) random forest model; (**d**) XGBoost model; (**e**) 1D-CNN model; and (**f**) proposed DNN model.

**Figure 10:**
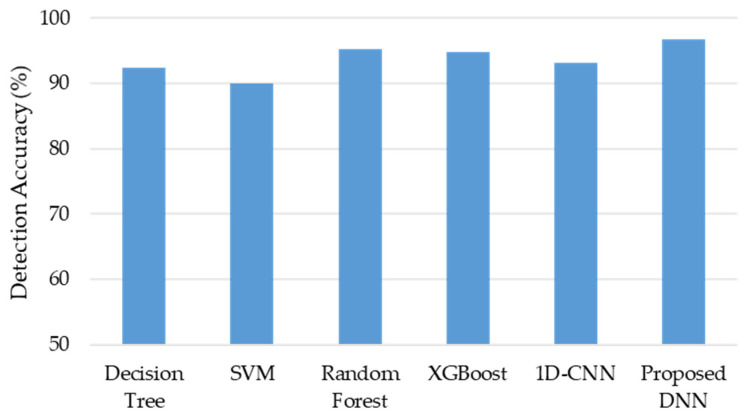
Classification percentage accuracy from different models.

Furthermore, the performance of the developed DNN was evaluated by comparing it with other machine learning models such as the decision tree model, SVM model, random forest model, XGBoost model, and 1D-CNN model. The same training dataset and testing datasets were used for all models and we utilized the default hyperparameters when training each model for a fair comparison. The diagnosis confusion matrix and the diagnosis accuracy of each algorithm for the testing are illustrated in Figure 9 and Figure 10, respectively. They show that the proposed DNN algorithm outperformed the other models; particularly, the fake tracking of the AGV could be detected with an accuracy of 91.72% and the accuracy of the real tracking reached 98.92%. This was because the DNN model was more efficient at learning complex features contained within the fake data. The classification accuracy results from the different models are listed in Table 2. The experimental results indicated that the proposed DNN model provided the best performance followed by the supported vector machine model (SVM) with an accuracy of 94%. The random forest, one dimension convolutional neural network (1D-CNN), extreme gradient boosting (XGBoost), and decision tree models had the worst results with classification accuracies of approximately 93.73%, 92%, 91.87, and 90%, respectively. Note that the performances of the machine learning models were assessed by:

(5) $$accuracy=\frac{TP+TN}{TP+FP+TN+FN}$$

where *TP* is true positive, *TN* is true negative, *FP* is false positive, and *FN* is false negative.

**Figure 11:**
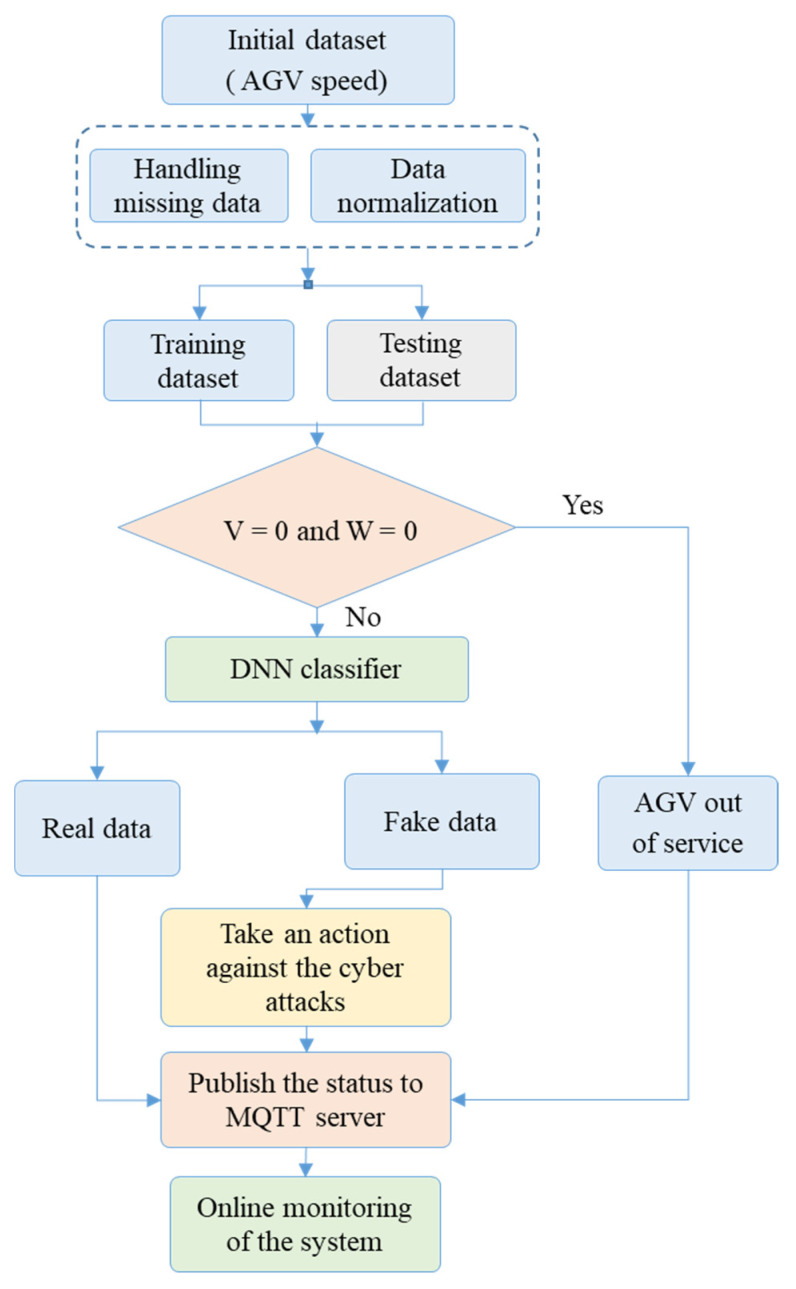
The flowchart of the proposed strategy based on the DNN for providing cybersecurity and online monitoring of the AGV.

Figure 11 summarizes the proposed strategy based on the DNN for providing cybersecurity and the online monitoring of the AGV. It is shown from this figure that the proposed DNN could analyze the data of the AGV and check if these data were real or fake, then publish the status of the AGV on the real-time IoT dashboard. The next subsections present various scenarios that validated the proposed IoT architecture.

### 4.3. Scenario 1: Real Tracking for AGVs

**Figure 12:**
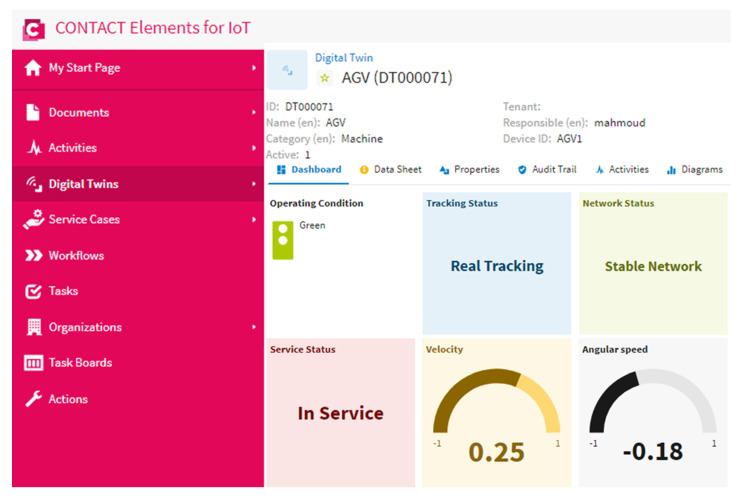
The AGV and network status in the case of a real data transfer and stable internet.

This test case was created to confirm the performance of the suggested DNN to indicate the real status of the AGV. Figure 12 shows the attitude of the AGV and the network condition on the dashboard of the proposed IoT platform. This figure demonstrates that the proposed DNN could recognize the real status of the AGV effectively. The suggested IoT platform could present the attitude of the AGV and the network condition in a more clarified presentation. Furthermore, the operating condition indicator was green, which confirmed that there was no fault and that the AGV in service and the internet network were reliable.

### 4.4. Scenario 2: Out of Service State

**Figure 13:**
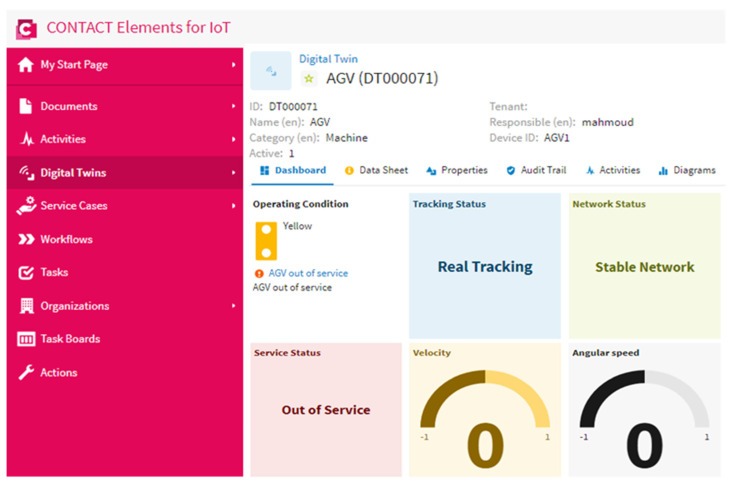
The AGV and network status in the case of an out of service state and a stable internet.

This scenario was performed to visualize the AGV status when it was out of service due to faults, charging, or maintenance. The out of service state was recognized when the velocity and the angular speed of the AGV were equal to zero. Figure 13 presents the AGV status when it was out of service. It is clear from this figure that the velocity and the angular speed of the AGV were equal to zero and the network was stable. The operating condition indicator varied to a yellow color to remind the operator that the AGV was out of service. This scenario confirmed that the suggested IoT architecture could record when the AGV was in an out of service state, which enhanced the decision-making.

### 4.5. Scenario 3: Cyber Attack and Fake Tracking

**Figure 14:**
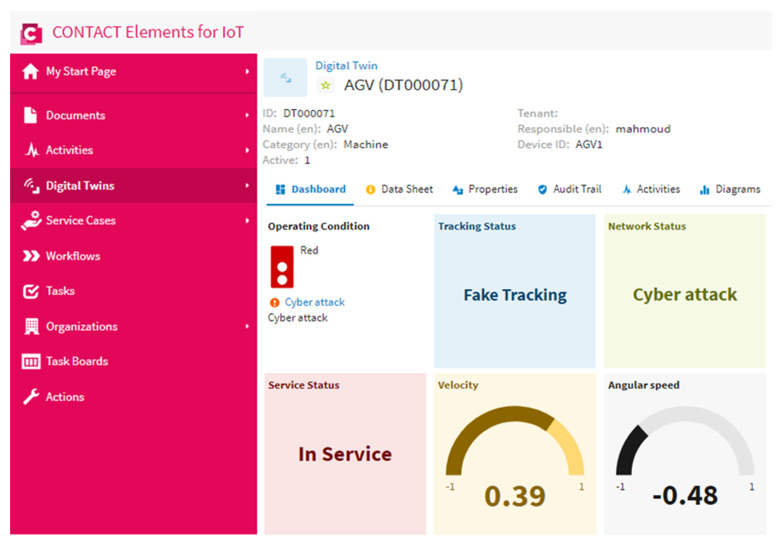
The AGV and network status in the case of a cyber attack and an unstable internet.

The cyber attack demonstrated the biggest issue against the enforcement of an IoT infrastructure. This scenario was performed to assert the distinction of the suggested IoT architecture based on the DNN to detect a cyber attack within the internet network. Figure 14 shows the AGV and the network status when there was a cyber attack. It is obvious from this figure that the transferred data regarding the velocity and the angular speed of the AGV were fake, which meant that the network was unstable. The operating condition indicator varied to a red color to remind the operator that the network was unstable. This scenario confirmed that the proposed IoT architecture based on the DNN could recognize a cyber attack on the internet network effectively and visualize the status in a clearer dashboard.

### 4.6. Discussion

The following points summarize the main discussions of the previous test scenarios:

- The normal state of the AGV was recognized and visualized effectively by the proposed IoT architecture based on the DNN, as presented in scenario 1. Furthermore, the network status was presented in the IoT dashboard beside the AGV speed to confirm the real tracking of the AGV.
- The out of service state of the AGV due to any fault, maintenance, and charging were presented in scenario 2. The out of service state was detected when the AGV speed was equal to zero. The proposed IoT architecture recorded and presented this state effectively on the IoT dashboard. Furthermore, the CONTACT Elements for the IoT performed an alarm by changing the light color of the operating condition indicator to a yellow color to indicate to the operator about the outage of the AGV from the service to support the decision-making.
- The instability of the network due to a cyber attack was detected successfully by the proposed IoT architecture based on the DNN, as clarified in scenario 3. This abnormal state was presented in a clearer way on the dashboard of the IoT platform. The light color of the operating condition indicator changed to a red color as an alarm to indicate to the operator that there was a cyber attack on the internet network.

## 5. Conclusions

This paper introduced a developed IoT architecture based on a DNN to visualize the status of an AGV. The study was performed to provide low cost cyber attack detection for real-time AGV monitoring. The development of online cyber attack suppression and intelligent IoT systems for AGVs is a key contributor to improving productivity in modern manufacturing. The performance of the proposed DNN was compared with other learning algorithms from the literature. The AGV status was visualized on a developed IoT platform called CONTACT Elements for IoT based on the suggested IoT architecture. The proposed IoT architecture based on the DNN effectively identified and simulated the normal state of the AGV. In contrast, the proposed IoT architecture detected the out of service condition and essentially tracked and monitored it on the dashboard of the IoT network whilst also creating an alert to notify the customer of the AGV outage. Most significantly, the suggested IoT architecture built on the DNN easily detected network instability caused by cyber attacks. Various test scenarios were performed to assert the superiority of the suggested IoT architecture based on the DNN to recognize the cyber attack and visualize the AGV status. The results emphasized the effectiveness of the proposed IoT architecture based on the DNN to provide secure monitoring for the AGV status, which enhances decision-making and can be applied to different applications. Furthermore, the provided information from the proposed IoT architecture based on the DNN regarding the cyber attack could help to design a robust controller against the problem of cyber attacks in future works.

## Figures and Tables

**Table 1:** DNN model parameters of each layer.

Layer (Type)	Output Shape	Number of Parameters
Input layer	(1, 4)	-
Hidden layer_1	(None, 32)	160
Hidden layer_2	(None, 64)	2112
Hidden layer_3	(None, 128)	8320
Hidden layer_4	(None, 32)	4128
Output layer	(None, 2)	66
Total parameters: 14,786		
Trainable parameters: 14,786		
Non-trainable parameters: 0		

**Table 2:** The classification accuracy from various algorithms.

Methods	Decision Tree	SVM	Random Forest	XGBoost	1D-CNN	Proposed DNN
Detection Accuracy (%)	92.43	90	95.23	94.81	93.13	96.77

## Data Availability

The data presented in this study are available on request from the corresponding author.

## References

[1] Zhang Y., Guo L., Gao B., Qu T., Chen H. (2020). Deterministic Promotion Reinforcement Learning Applied to Longitudinal Velocity Control for Automated Vehicles. IEEE Trans. Veh. Technol..

[2] Zhang Z., Guo Q., Chen J., Yuan P. (2018). Collision-free route planning for multiple AGVs in an automated warehouse based on collision classification. IEEE Access.

[3] Su S., Dai H., Cheng S., Chen Z. (2020). Improved Magnetic Guidance Approach for Automated Guided Vehicles by Error Analysis and Prior Knowledge. IEEE Trans. Intell. Transp. Syst..

[4] Zhu S., Gelbal S.Y., Aksun-Guvenc B., Guvenc L. (2019). Parameter-Space Based Robust Gain-Scheduling Design of Automated Vehicle Lateral Control. IEEE Trans. Veh. Technol..

[5] Goli A., Tirkolaee E.B., Aydin N.S. (2021). Fuzzy Integrated Cell Formation and Production Scheduling Considering Automated Guided Vehicles and Human Factors. IEEE Trans. Fuzzy Syst..

[6] Oyekanlu E.A., Smith A.C., Thomas W.P., Mulroy G., Hitesh D., Ramsey M., Kuhn D.J., Mcghinnis J.D., Buonavita S.C., Looper N.A. (2020). A Review of Recent Advances in Automated Guided Vehicle Technologies: Integration Challenges and Research Areas for 5G-Based Smart Manufacturing Applications. IEEE Access.

[7] Hrušecká D., Lopes R.B., Juřičková E. (2019). Challenges in the introduction of AGVS in production lines: Case studies in the automotive industry. Serb. J. Manag..

[8] Al-Turjman F., Lemayian J.P. (2020). Intelligence, security, and vehicular sensor networks in internet of things (IoT)-enabled smart-cities: An overview. Comput. Electr. Eng..

[9] AlZubi A.A., Alarifi A., Al-Maitah M., Alheyasat O. (2021). Multi-sensor information fusion for Internet of Things assisted automated guided vehicles in smart city. Sustain. Cities Soc..

[10] Wan J., Tang S., Hua Q., Li D., Liu C., Lloret J. (2018). Context-Aware Cloud Robotics for Material Handling in Cognitive Industrial Internet of Things. IEEE Internet Things J..

[11] Yao F., Keller A., Ahmad M., Ahmad B., Harrison R., Colombo A.W. Optimizing the scheduling of autonomous guided vehicle in a manufacturing process. Proceedings of the 2018 IEEE 16th International Conference on Industrial Informatics (INDIN), IEEE.

[12] Bhamare D., Zolanvari M., Erbad A., Jain R., Khan K., Meskin N. (2020). Cybersecurity for industrial control systems: A survey. Comput. Secur..

[13] Kravchik M., Shabtai A. Detecting cyber attacks in industrial control systems using convolutional neural networks. Proceedings of the 2018 Workshop on Cyber-Physical Systems Security and PrivaCy.

[14] Han S., Xie M., Chen H.H., Ling Y. (2014). Intrusion detection in cyber-physical systems: Techniques and challenges. IEEE Syst. J..

[15] Pasqualetti F., Dörfler F., Bullo F. Cyber-physical attacks in power networks: Models, fundamental limitations and monitor design. Proceedings of the 2011 50th IEEE Conference on Decision and Control and European Control Conference, IEEE.

[16] Teixeira A., Pérez D., Sandberg H., Johansson K.H. Attack models and scenarios for networked control systems. Proceedings of the 1st International Conference on High Confidence Networked Systems.

[17] Mitchell R., Chen I.R. (2014). A survey of intrusion detection techniques for cyber-physical systems. ACM Comput. Surv. (CSUR).

[18] Elsisi M., Mahmoud K., Lehtonen M., Darwish M.M. (2021). Reliable Industry 4.0 Based on Machine Learning and IoT for Analyzing, Monitoring, and Securing Smart Meters. Sensors.

[19] Bligh-Wall S. (2017). Industry 4.0: Security imperatives for IoT—Converging networks, increasing risks. Cyber Secur. Peer-Rev. J..

[20] Preuveneers D., Ilie-Zudor E. (2017). The intelligent industry of the future: A survey on emerging trends, research challenges and opportunities in Industry 4.0. J. Ambient. Intell. Smart Environ..

[21] Benzaoui N., Gonzalez M.S., Rivera M.V., Estaran J.M., Mardoyan H., Lautenschlaeger W., Gebhard U., Dembeck L., Pointurier Y., Bigo S. DDN: Deterministic dynamic networks. Proceedings of the 2018 European Conference on Optical Communication (ECOC), IEEE.

[22] Redana S., Bulakci Ö., Zafeiropoulos A., Gavras A., Tzanakaki A., Albanese A., Kousaridas A., Weit A., Sayadi B., Jou B.T. (2019). 5G PPP Architecture Working Group: View on 5G Architecture.

[23] IoT Platform for Digital Business Models. CONTACT Software. https://www.contact-software.com/en/.

[24] Tran M.-Q., Elsisi M., Mahmoud K., Liu M.-K., Lehtonen M., Darwish M.M.F. (2021). Experimental Setup for Online Fault Diagnosis of Induction Machines via Promising IoT and Machine Learning: Towards Industry 4.0 Empowerment. IEEE Access.

[25] Elsisi M., Tran M.-Q., Mahmoud K., Lehtonen M., Darwish M.M.F. (2021). Deep Learning-Based Industry 4.0 and Internet of Things towards Effective Energy Management for Smart Buildings. Sensors.

[26] Schmidhuber J. (2015). Deep learning in neural networks: An overview. Neural Netw..

[27] Ohn I., Kim Y. (2019). Smooth Function Approximation by Deep Neural Networks with General Activation Functions. Entropy.

[28] Zhang J. (2019). Gradient descent based optimization algorithms for deep learning models training. arXiv.

[29] Prasad S. (2020). Counteractive control against cyber-attack uncertainties on frequency regulation in the power system. IET Cyber-Phys. Syst. Theory Appl..

[30] Pasqualetti F., Dörfler F., Bullo F. (2013). Attack detection and identification in cyber-physical systems. IEEE Trans. Autom. Control.

[31] Habibi M.R., Sahoo S., Rivera S., Dragičević T., Blaabjerg F. (2021). Decentralized coordinated cyber-attack detection and mitigation strategy in DC microgrids based on artificial neural networks. IEEE J. Emerg. Sel. Top. Power Electron..

